# Complications and hospitalization costs in patients with hypothyroidism following total hip arthroplasty

**DOI:** 10.1186/s13018-023-04057-0

**Published:** 2023-08-04

**Authors:** Yuanyuan Huang, Yuzhi Huang, Yuhang Chen, Qinfeng Yang, Binyan Yin

**Affiliations:** 1 School of Health, Dongguan Polytechnic, Dongguan, 523000 Guangdong China; 2 Department of Anesthesiology, Nanfang Hospital, Southern Medical University, Guangzhou, 510515 Guangdong China; 3 Department of Orthopaedic Surgery, Division of Orthopaedics, Nanfang Hospital, Southern Medical University, Guangzhou, 510515 Guangdong China; 4 Guangdong Medical University, Dongguan, 523109 Guangdong China; 5 Department of Orthopedic Surgery, The First People’s Hospital of Foshan, Foshan, 528000 Guangdong China

**Keywords:** Total hip arthroplasty, Hypothyroidism, Nationwide inpatient sample, Outcomes, Comorbidity

## Abstract

**Background:**

Hypothyroidism is a common disease in the US population. The impact of hypothyroidism on perioperative complications of total hip arthroplasty is poorly understood. To examine risk factors and hospitalization costs in patients with hypothyroidism after total hip arthroplasty (THA) using a large-scale sample national database.

**Methods:**

A case–control study was performed based on the national inpatient sample database from 2005 to 2014. With the use of propensity scores, patients with hypothyroidism were matched in a 1:1 ratio to those without hypothyroidism by age, gender, race, Elixhauser Comorbidity Index (ECI), and insurance type. Patient demographics, postoperative complications, length of stay (LOS), and hospital costs were compared between matched cohorts. Short-term complication rates after THA were compared using multivariate logistic analysis.

**Results:**

The proportion of patients with hypothyroidism receiving THA was 12.97%. Linear regression analysis yielded that patients with hypothyroidism receiving THA were more likely to have postoperative acute anemia (odds ratio = 1.15; 95% confidence interval = 1.12–1.18) and higher mean hospital costs compared to the non-hypothyroid cohort.

**Conclusions:**

This present study demonstrates that hypothyroid patients undergoing THA have a higher risk of short-term complications. Furthermore, it significantly increased the total cost of hospitalization, which deserves more attention from orthopedic surgeons.

## Introduction

Total hip arthroplasty (THA) is one of the most successful orthopedic procedures in the United States, with improved hip function and quality of life for patients with end-stage osteoarthritis [1–3]. In recent years, the aging population has increased THA demand significantly [4, 5], which places an enormous economic burden on the healthcare system [6–8]. There are enormous financial and personal costs associated with postoperative complications [9, 10]. Therefore, to reduce costs and postoperative complication rates, recent efforts have focused on predicting independent risk factors and adverse clinical outcomes.

Thyroid hormones play a crucial role in affecting bone and cartilage [11, 12]. Studies have shown that thyroid disease is associated with an increased risk of osteoporosis and fractures [13, 14]. Among people with joint disease, thyroid disease is more prevalent than in the general population, with a prevalence of 18% [15]. Furthermore, Hypothyroidism is associated with an increased risk of Periprosthetic joint infection (PJI) [16, 17].

Although hypothyroidism may increase the risk of postoperative complications, it is not an absolute contraindication to THA. To our knowledge, the impact of hypothyroidism on in-hospital complications and hospital costs after THA has not been previously assessed by large-scale data analysis. Therefore, the study evaluated the influence of hypothyroidism on in-hospital complication rates and hospital costs in patients undergoing THA with the use of the National Inpatient Sample (NIS) database. This study assessed: (1) patient morbidity in hypothyroidism patients undergoing THA, (2) demographic characteristics of patients, (3) surgical and medical complications, (4) the length of hospital stay (LOS) and total hospital costs in patients with hypothyroidism receiving THA.

## Methods

### Data source

The National Inpatient Sample (NIS) database was employed as the source of data. Since 1998 this database has been considered the largest all-payer inpatient database in the U.S, NIS database collected stratified samples from over 1000 hospitals, accounting for approximately 20% of U.S. inpatient admissions each year [17–19]. The NIS database was developed as a part of the Healthcare Billing and Utilization Project (HCUP). It was sponsored by the Agency for Healthcare Research and Quality (AHRQ). The data used in the research were deidentified, hence protecting privacy and confidentiality issues and making the current study exempt.

### Data collection

The NIS database was used to collect information on patients undergoing THA surgery from 2005 to 2014. The data collected included demographics, diagnoses and procedures defined by the International Classification of Disease, 9th edition (ICD-9) diagnosis and procedure codes, LOS, hospital costs, and insurance type. ICD-9-CM procedure codes were used to identify THA patients (81.51) (n = 593,045). Patients receiving THA were then divided into two groups, those with hypothyroidism and those without hypothyroidism (ICD-9-CM codes were 244.0/244.1/244.2/244.3/244.8).

Patients undergoing THA were examined for perioperative complications using the ICD-9-CM diagnosis code. When one or more surgical or medical complications have happened, the phrase “any complication” was used. Medical complications included acute postoperative anemia, thrombocytopenia, intubation, acute renal failure (ARF), acute myocardial infarction (AMI), pneumonia, pulmonary embolism (PE), stroke, postoperative delirium (PD), urinary tract infection (UTI), deep vein thrombosis/thrombophlebitis (DVT), sepsis, postoperative shock, and blood transfusion. Surgical complications included wound infection, wound dehiscence, hematoma, irrigation and debridement, injury to peripheral nerve of lower limb, periprosthetic joint infection, mechanical loosening of prosthetic joint, dislocation of prosthetic joint, peri-prosthetic fracture around prosthetic [15, 18, 20]. The LOS was calculated as days from hospital admission to discharge, and the total cost was also collected from the database.

**Fig. 1:**
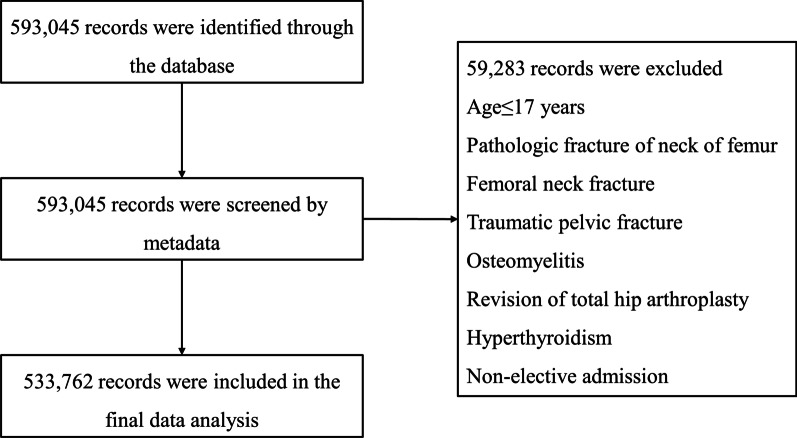
Flow diagram of complications and hospitalization costs in patients with hypothyroidism following total hip arthroplasty

As shown in Fig. 1, patients with pathologic fracture of neck of femur (733.14), femoral neck fracture (820.0–820.9), traumatic pelvic fractures (808.0/808.1), osteomyelitis (730.25), revision THA (81.53), hyperthyroidism (242.0/242.1/242.2/242.3/242.9), age less than 18 years, and non-elective admission (n = 59,283) were excluded. A total of 69,249 hypothyroidism patients who underwent THA from 2005 to 2014 were detected (Table 1).

**Table 1:** Demographics of total hip arthroplasty patients with and without hypothyroidism

Variable	Hypothyroidism	No hypothyroidism	P value
N	69,249	464,513	–
*Total comorbidity rate*, %	12.97%		
Age in years, mean	69	65	< 0.0001
*Age distribution*, n (%)			< 0.0001
18–44	1264(1.83%)	26,331(5.67%)	
45–64	22,177(32.03%)	203,010(43.7%)	
65–74	22,975(33.18%)	133,575(28.76%)	
≥ 75	22,833(32.97%)	101,597(21.87%)	
*Gender*, n (%)			< 0.0001
Male	13,243(19.13%)	224,395(48.4%)	
Female	55,986(80.87%)	239,227(51.6%)	
*Race*, n (%)			< 0.0001
White	10,222(14.76%)	75,153(16.18%)	
Nonwhite	4842(6.99%)	55,066(11.85%)	
Missing	54,185(78.25%)	334,294(71.97%)	
*Elixhauser score*, n (%)			< 0.0001
0	9627(13.9%)	99,098(21.33%)	
1	20,642(29.81%)	150,015(32.3%)	
≥ 2	38,980(56.29%)	215,400(46.37%)	
*Insurance*, n (%)			< 0.0001
Medicare	44,347(64.13%)	230,543(49.72%)	
Medicaid	1318(1.91%)	17,253(3.72%)	
Private insurance	22,018(31.84%)	199,857(43.1%)	
Self-pay	277(0.4%)	3585(0.77%)	
No charge	50(0.07%)	645(0.14%)	
Other	1146(1.66%)	11,838(2.55%)	

### Patient demographics after propensity score matching

The Elixhauser Comorbidity Index (ECI), a rating system designed to estimate the influence of the underlying illness on clinical outcomes, particularly short-term mortality risk, was used to evaluate comorbidities [16, 21, 22]. To mitigate confounding demographic variables, propensity score matching was performed [20]. The ratio of hypothyroid to non-hypothyroid patients was 1:1 at matching, and following propensity score matching, the matched THA cohort had 69,136 hypothyroid THA patients and 69,136 non-hypothyroid THA patients (Table 2). This study randomly chose patients, resulting in two cohorts with the same age, gender, race, ECI, and checkout type distribution.

**Table 2:** Demographics of hypothyroidism and the matched cohort of total hip arthroplasty patients

Variable	Hypothyroidism	Matched controls	P value
N	69,136	69,136	
*AGE-group*, n (%)			0.4364
18–44	1260(1.82%)	1260(1.82%)	
45–64	22,130(32.01%)	22,214(32.13%)	
65–74	22,951(33.20%)	23,147(33.48%)	
≥ 75	22,795(32.97%)	22,515(32.57%)	
*Gender,* n (%)			0.4807
Male	13,228(19.13%)	13,124(18.98%)	
Female	55,908(80.87%)	56,012(81.02%)	
Race, n (%)			0.825
White	10,185(14.73%)	10,141(14.67%)	
Nonwhite	4838(7%)	4891(7.07%)	
Missing	54,113(78.27%)	54,104(78.26%)	
*Elixhauser score*, n (%)			0.4357
0	9594(13.88%)	9632(13.93%)	
1	20,613(29.82%)	20,806(30.09%)	
≥ 2	38,929(56.31%)	38,698(55.97%)	
*Insurance*, n (%)			0.5842
1 = Medicare	44,337(64.13%)	44,189(63.92%)	
2 = Medicaid	1318(1.91%)	1241(1.8%)	
3 = Private insurance	22,008(31.83%)	22,234(32.16%)	
4 = Self-pay	277(0.4%)	276(0.4%)	
5 = No charge	50(0.07%)	52(0.08%)	
6 = Other	1146(1.66%)	1144(1.65%)	

### Data analysis

All statistical analyses were carried out using the statistical software R version 3.5.3 (The R Foundation Inc.). In this cross-sectional analysis, the cohort consisted of patients with a diagnosis of THA. Surgical complications, postoperative complications, mortality, LOS, and total hospital costs were compared between patients with hypothyroid THA and matched control non-hypothyroid THA patients. Then, to account for possible confounders when comparing perioperative complications, mortality, LOS, and total hospital costs, 1:1 propensity score matching was performed based on age, gender, race, ECI, and insurance type. A multivariate logistic analysis was used to calculate the advantage ratio (AR) of surgical complications and postoperative complications during the as-needed period for patients with hypothyroidism compared to the non-hypothyroid matched cohort. To investigate the effect of interactions between hypothyroidism and comorbidities on LOS and hospitalization costs, parameter estimates of mean LOS and mean total hospitalization costs were calculated using linear regression analysis, describing the linear regression results in terms of percentage differences and reporting the odds ratio (OR) and 95% confidence interval (CI) using the formula (e^b^ − 1) × 100 [23], where b is the log-transformed dependent variable of parameter estimates.

## Results

### Prevalence of hypothyroidism in THA population

From 2005 to 2014, 533,762 THA instances were identified in the NIS database. Hypothyroidism incidence continuously grew (from 11.11 to 14.16%) and barely dropped in 2014 when compared to the previous year. The overall ten-year incidence of hypothyroidism following THA was 12.97%.

### Characteristics of hypothyroid patients

Patients with hypothyroidism were 4 years elder than those without (69 years vs. 65 years, P < 0.0001), and the prevalence was 11.10% higher in those over 75 years (32.97% vs. 21.87%, P < 0.0001).

### Perioperative surgical and medical complications

There was a non-significant difference in the probability of any complication, any surgical complication, or any medical complication in hypothyroid individuals compared to the matched sample (Table 3). When the specific medical complications were evaluated, hypothyroid patients have a 1.15 times higher risk of postoperative acute anemia compared with the matched cohort (OR = 1.15; 95% CI = 1.12–1.18; P < 0.0001) (Table 4). There was no statistically significant difference between hypothyroid and non-hypothyroid patients in the assessment of surgical complications (Table 5).

**Table 3:** Perioperative complications in patient undergoing total hip arthroplasty with or without hypothyroidism

Parameter	Hypothyroidism^a^	Matched controls	P value
Any complication	0.9167(0.7923–1.0608)	1.00	0.2430
Any medical complication	1.1690(1.0119–1.3506)	1.00	0.0339
Any surgical complication	0.9394(0.8713–1.0127)	1.00	0.1029

^a^Data are presented as odds ratio and (95% confidence intervals)

**Table 4:** Multivariate logistic regression analysis of medical complications in patients undergoing total hip arthroplasty with and without hypothyroidism

Parameter	Univariate analysis, % (n)			Multivariate logistic regression	
	Hypothyroidism	Matched controls	*P* value	OR (95% CI)	*P* value
Acute postoperative anemia	20,492(29.64%)	18,665(27%)	< 0.0001	1.1467(1.1191–1.1749)	< 0.0001
Thrombocytopenia	1200(1.74%)	1,096(1.59%)	0.0302	1.0638(0.9790- 1.1559)	0.1444
Intubation	≤ 10^a^(0.01%)	13(0.02%)	0.3827	0.6040(0.2481- 1.4706)	0.2668
Acute renal failure	1266(1.83%)	1092(1.58%)	0.0003	1.1400(1.0494–1.2383)	0.0019
Acute myocardial infarction	498(0.72%)	530(0.77%)	0.3318	0.9330(0.8248–1.0553)	0.2696
Pneumonia	292(0.42%)	369(0.53%)	0.003	0.7872(0.6736–0.9198)	0.0026
Pulmonary embolism	116(0.17%)	118(0.17%)	0.9478	0.9975(0.7702–1.2919)	0.9851
Stroke	34(0.05%)	40(0.06%)	0.561	0.8593(0.5432–1.3594)	0.5170
Postoperative delirium	729(1.05%)	693(1%)	0.3508	1.0392(0.9357–1.1542)	0.4725
Urinary tract infection	2130(3.08%)	2035(2.94%)	0.1391	1.0391(0.9766–1.1056)	0.2260
Deep vein thrombosis	154(0.22%)	170(0.25%)	0.4041	0.8956(0.7188–1.1159)	0.3258
Sepsis	46(0.07%)	65(0.09%)	0.0874	0.7171(0.4871–1.0559)	0.0921
Postoperative shock	35(0.05%)	28(0.04%)	0.4496	1.2247(0.7411–2.0237)	0.4290
Blood transfusion	17,230(24.92%)	17,273(24.98%)	0.7941	0.9593(0.9353–0.9839)	0.013

*OR* odds ratio; *CI* confidence interval

^a^Cells with frequency < 11 were suppressed due to the protection of patient privacy

**Table 5:** Multivariate logistic regression analysis of surgical complications in patients undergoing total hip arthroplasty with and without hypothyroidism

Parameter	Univariate analysis, % (n)			Multivariate logistic regression	
	Hypothyroidism	Matched controls	*P* value	OR (95% CI)	*P* value
Wound infection	67(0.1%)	59(0.09%)	0.5327	1.1488(0.8092–1.6310)	0.4378
Wound dehiscence	≤ 10^a^(0.01%)	≤ 10^a^(0.01%)	0.6056	0.7039(0.2488–1.9917)	0.5082
Hemorrhage/seroma/hematoma	577(0.83%)	594(0.86%)	0.6387	0.9798(0.8730–1.0997)	0.7289
Irrigation and debridement	68(0.1%)	83(0.12%)	0.2543	0.8333(0.6030–1.1518)	0.2695
Injury to peripheral nerve of lower limb	22(0.03%)	34(0.05%)	0.1415	0.6518(0.3811–1.1146)	0.1179
PJI	40(0.06%)	39(0.06%)	1	1.0433(0.6707–1.6229)	0.8508
Mechanical loosening	50(0.07%)	62(0.09%)	0.2984	0.8155(0.5610–1.1855)	0.2852
Dislocation	135(0.2%)	156(0.23%)	0.2405	0.8699(0.6905–1.0959)	0.2368
Peri-prosthetic fracture	75(0.11%)	74(0.11%)	1	1.0371(0.7510–1.4321)	0.8250
Other prosthetic-related complication	920(1.33%)	978(1.41%)	0.1877	0.9426(0.8608–1.0321)	0.2013

^a^Cells with frequency < 11 are suppressed due to the protection of patient privacy

### Postoperative outcomes in patients with hypothyroidism

Compared with the matched cohort, hypothyroidism significantly increased the average total hospital cost by $1378.48 ($51,306.89 vs. $49,928.41, 95% CI, Ig1.0368–Ig1.4824, P < 0.0001), and the mean LOS for patients with hypothyroidism was 3.31 days, while the non-hypothyroid patients had a mean LOS of 3.34 days (P = 0.0088), and linear regression analysis yielded a mean LOS of 2.3739% less (95% CI, − 4.11 to 0.60, P = 0.0088) (Table 6).

**Table 6:** Hospital outcomes for patients undergoing total hip arthroplasty with and without hypothyroidism

Parameter	Hypothyroidism	Matched controls	*P* value
*Lengths of stay (days)*			
Mean (SD)	3.31(1.57)	3.34(1.83)	0.0088
Percent difference (95% CI)	− 2.3739(− 4.1120 to − 0.6042)	0.00	0.0088
*Charges* ($)			
Mean (SD)	51,306.89(27,302.05)	49,928.41(27,856.77)	< 0.0001
Percent difference (Ig(95% CI))	1.2593(1.0368–1.4824)	0.00	< 0.0001
*Mortality rate* (%)			
Rate	0.08	0.1	0.374
Percent difference (95% CI)	− 15.9737(− 40.7432 to 19.1497)	0.00	0.3287

*OR* odds ratio; *CI* confidence interval

## Discussion

The present study aims to determine whether hypothyroidism conferred an increased risk of complications in patients following THA. According to our results, hypothyroidism may not be a risk factor for increased short-term complication rates in patients receiving THA, and a paucity of studies have focused on short-term complications after surgery. Patients receiving THA did not show a significant interaction between complications and hypothyroidism. Nevertheless, the present study revealed that hypothyroidism significantly increased hospitalization costs. An explanation for this finding may attribute to additional perioperative managements and laboratory tests for hypothyroidism.

In agreement with Tan et al. [15], 12.97% of patients who received THA had hypothyroidism before surgery. However, in this study, the occurrence of perioperative complications in patients with hypothyroidism undergoing THA is lower than that in patients with hypothyroidism receiving other joint replacements [15, 16]. This may be related to well-established perioperative management, with reports that reversing hypothyroidism before joint arthroplasty did not increase surgical risk [24]. Whether hypothyroid patients receiving THA received appropriate hypothyroidism preoperatively was not identified in this study. But compared to the general population, patients with hypothyroidism are more likely to undergo orthopedic surgery, likely because thyroid hormone deficiency can cause degenerative diseases [25, 26].

We found that hypothyroidism was associated with acute postoperative anemia, while postoperative anemia has been reported as a common complication in patients with hypothyroidism undergoing joint replacement surgery [15, 16]. Therefore, some studies aimed to reduce the incidence of perioperative complications in patients with hypothyroidism by adjusting thyroid hormone levels [27]. Clinical studies also indicated that hypothyroidism led to an elevated risk of infection and delayed wound healing [15]. In this regard, preoperative management should be considered to suppress metabolic abnormalities caused by hypothyroidism. However, studies have found that overtreatment of hypothyroidism with levothyroxine can be a negative factor in fracture risk and bone metabolism, and no study has reported whether the correct balance of hypothyroidism can reverse the negative effects on bone [28, 29]. According to Ling et al. [30], biochemical hyperthyroidism was considered to be associated with early complications in patients undergoing surgery for hip fractures. Accounting for widespread systemic manifestations, thyroid hormones have influenced on many different tissues, and we have found that hypothyroidism were in association with several complications following THA. However, there was no ICD codes or existed variable in the NIS database, so we were unable to detected the thyroid function test of these population, and further study to discuss the impact of preoperative thyroid function management on patients undergoing THA was merited.

Anemia is a common complication of hypothyroidism and may be associated with bone marrow stimulation suppression, decreased erythropoietin production, nutritional deficiencies, and coexisting disorders [31], suggesting a complex mechanism for the development of postoperative anemia due to hypothyroidism. Studies have shown that hypothyroidism makes the hemostatic system in a hyperfibrinolytic and hypocoagulable state [32, 33]. Furthermore, clinical trials have verified that postoperative hemoglobin (Hb) decline and total perioperative bleeding are higher in hypothyroid patients than in non-hypothyroid patients [34]. Hypothyroidism leads to disruption of coagulation homeostasis in patients, and abnormal bleeding volume as well as delayed wound healing may be the main cause of acute anemia [15, 33, 34].

Anemia is a common complication after THA that increases the incidence of adverse clinical outcomes [35, 36]. Previous studies have shown that serious preoperative anemia in those with hip diseases reflected potentially poorer physical function and nutritional deficiency, leading to slower postoperative recovery, and blood transfusion shew limited effect. As hypothyroidism might be a risk factor in developing anemia, surgeons are suggested to improve the levels of thyroid function rather than transfuse blood preoperatively [37]. Some clinical trials have shown that preoperative adjustment of serum TSH and T4 levels in hypothyroid patients resulted in a non-significant difference in transfusion rates compared to controls, suggesting that perioperative blood management and anti-hyperthyroid treatment can mitigate the adverse consequences of bleeding [38]. Moreover, the application of tranexamic acid (TXA) significantly reduced the magnitude of postoperative Hb decline and the chance of patients requiring allogeneic blood transfusion [38–41]. As we all know, a lab test is an important indicator to diagnose anemia. Whereas, in recent years, there has been controversy over whether the laboratory examination of patients after THA is complete [42, 43]. Some studies have found that most laboratory tests do not affect postoperative management and the most common abnormalities are anemia and hypoproteinemia, which require intervention, while other abnormalities often do not require further treatment, suggesting the need for postoperative laboratory tests based on the patient's preoperative condition [42, 44, 45].

There are some limitations in the present study. As with other large databases studies, there may be coding discrepancies and data entry errors when performing retrospective analyses [46]. As a result, our study might underestimate the incidence of postoperative complications. On the other hand, the data were obtained by screening existed variables in the NIS database and ICD-9 codes, and unfortunately, there was no variable or code documenting the level of fT3, fT4, and thyroid stimulating hormone, making thyroid function undetectable in our study. Likewise, preoperative management on thyroid function could hardly be observed. Therefore, thyroid dysfunction merits further prospective studies to determine the influence of thyroid function management on postoperative outcomes. Another limitation of the study was the lack of a clear length of time to distinguish between short-term and long-term complications, such as 30 and 90 days [46–48]. Nonetheless, the present study is that only short-term outcomes were evaluated, so the long-term effects of cup or stem loosening could not be well assessed.

## Conclusions

This study shows that hypothyroidism increases the risk of acute postoperative anemia and hospital costs in patients with THA. Improving patient thyroid function and refining appropriate blood management strategies can help patients reduce the incidence of adverse clinical outcomes. Further prospective studies need to be completed to examine whether improving thyroid function reduces hospitalization costs for patients. The results of this study may improve surgeons’ ability to counsel patients preoperatively about the specific risks associated with hypothyroidism and THA.
